# Supplementary figures and images for: Global, regional, and national burden of cataract: A comprehensive analysis and projections from 1990 to 2021
Source: PLoS One. 2025 Jun 23;20(6):e0326263. doi: 10.1371/journal.pone.0326263 (PMC12185006; doi:10.1371/journal.pone.0326263)

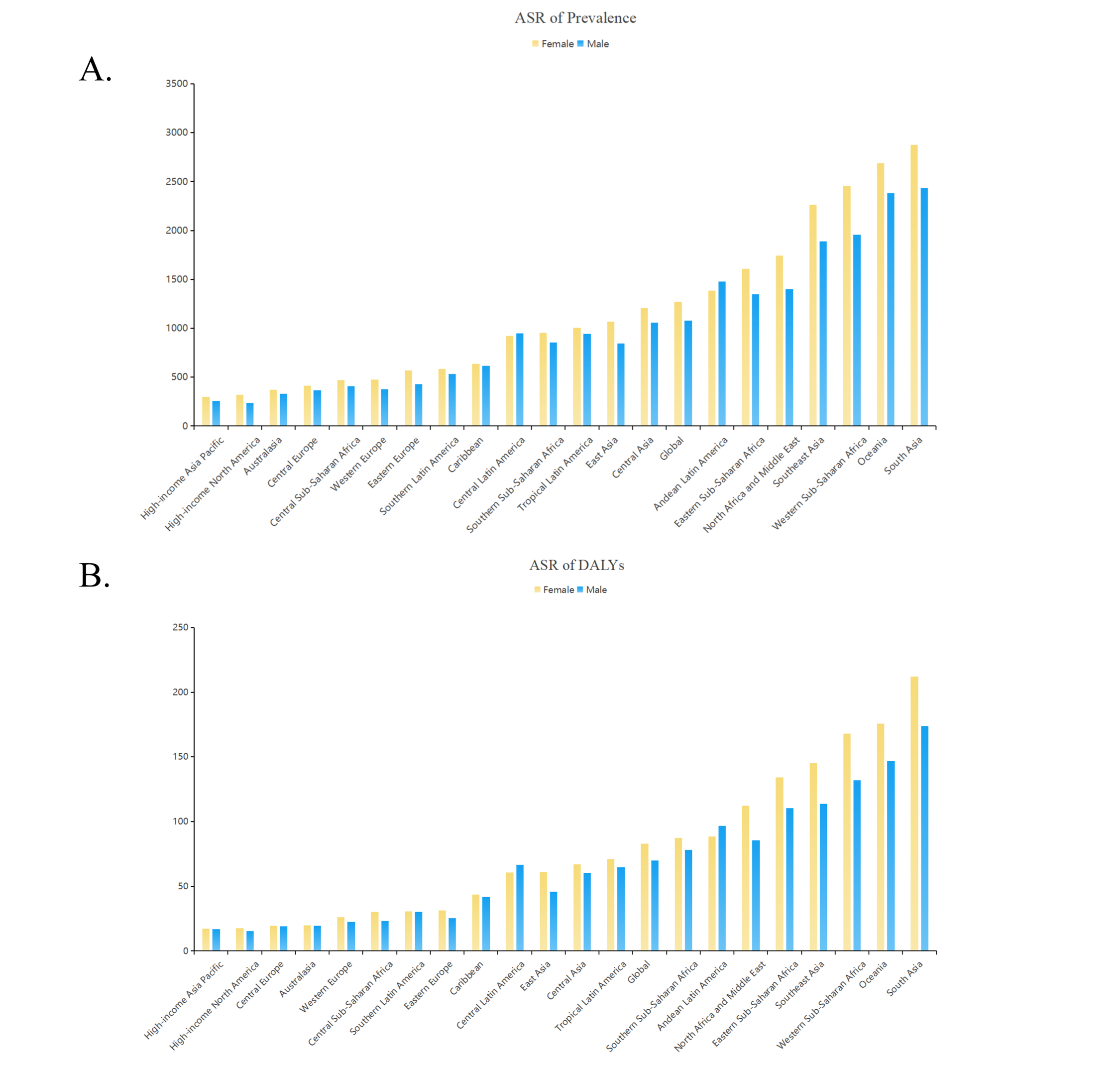

Supplement: S1 Fig — (TIF) [file pone.0326263.s001.tif]

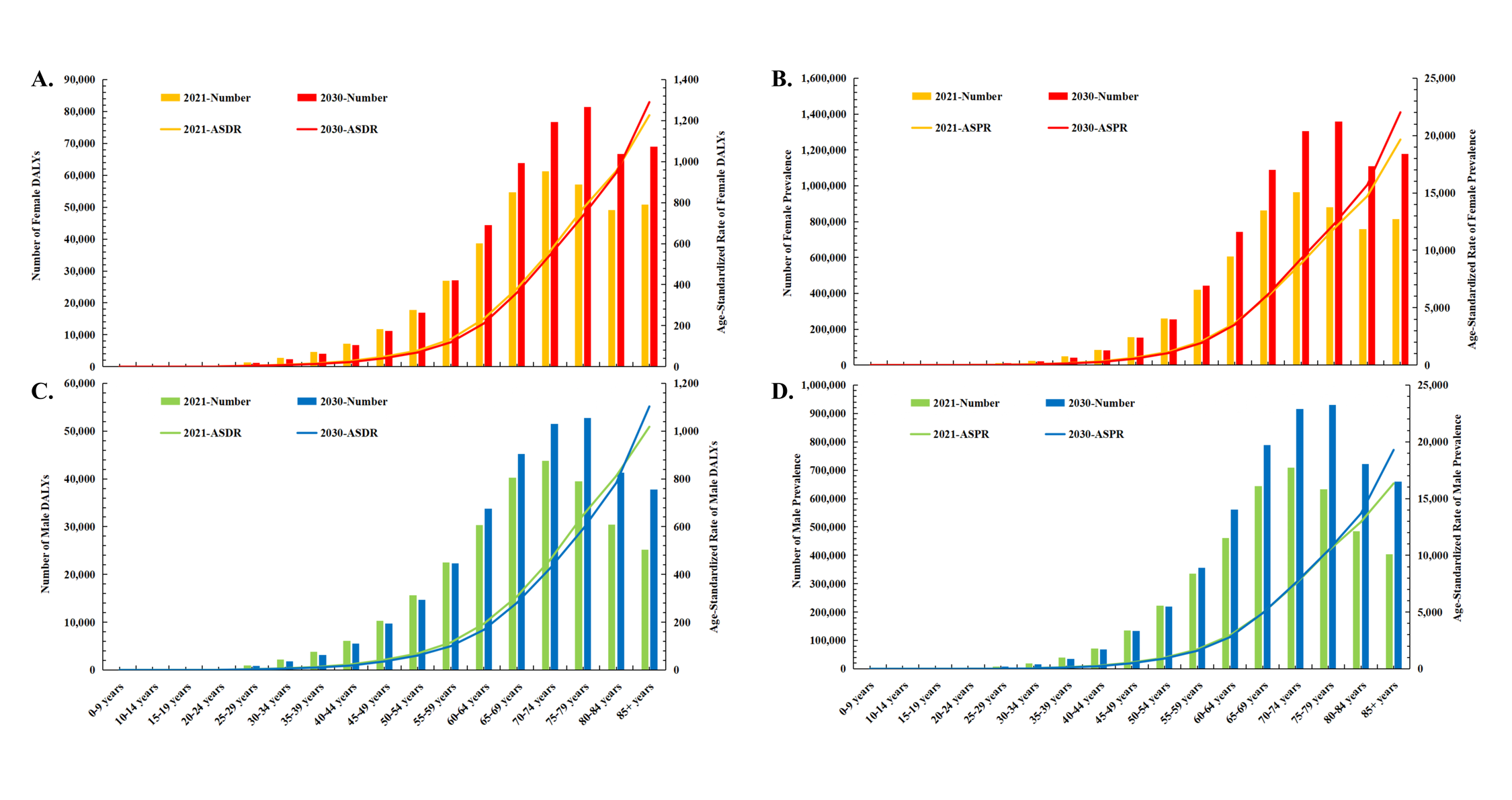

Supplement: S2 Fig — (TIF) [file pone.0326263.s002.tif]
